# The consequences of obesity on trauma, emergency surgery, and surgical critical care

**DOI:** 10.1186/1749-7922-1-27

**Published:** 2006-09-06

**Authors:** Carlos VR Brown, George C Velmahos

**Affiliations:** 1 Department of Surgery, Division of Trauma and Critical Care, University of Southern California Keck School of Medicine, Los Angeles County/University of Southern California Medical Center, Los Angeles, California, USA; 2 Department of Surgery, Division of Trauma, Emergency Surgery, and Critical Care, Harvard Medical School, Massachusetts General Hospital, Boston, Massachusetts, USA

## Abstract

The era of the acute care surgeon has arrived and this "new" specialty will be expected to provide trauma care, emergency surgery, and surgical critical care to a variety of patients arriving at their institution. With the exception of practicing bariatric surgeons, many general surgeons have limited experience caring for obese patients. Obese patients manifest unique physiology and pathophysiology, which can influence a surgeon's decision-making process. Following trauma, obese patients sustain different injuries than lean patients and have worse outcomes. Emergency surgery diseases may be difficult to diagnose in the obese patient and obesity is associated with increased complications in the postoperative patient. Caring for an obese patient in the surgical ICU presents a distinctive challenge and may require alterations in care. The following review should act as an overview of the pathophysiology of obesity and how obesity modifies the care of trauma, emergency surgery, and surgical critical care patients.

## Background

The prevalence of obesity in this country continues to increase at an alarming rate. In fact, recent figures estimate that almost one third of adults in this country can be classified as obese [1]. While obesity remains mostly a prevention and public health issue, it is becoming an everyday part of daily surgical practice. As we progress in the new age of the acute care surgeon, the practicing surgeon will need a core understanding of the pathophysiology of obesity and how obesity impacts the care of critically ill and injured surgical patients.

## Pathophysiology

Obesity influences a variety of organ systems altering the expected physiologic response to injury and illness. Obese patients may have diagnosed (or undiagnosed) physiologic abnormalities leading to compromise in cardiovascular, pulmonary, or metabolic physiology. Obesity leads to both structural and functional abnormalities of the cardiovascular system. In general, obese individuals will have an increased circulating blood volume as well as in increase in cardiac output [2,3], thought to be the result of increased oxygen demand from extra body tissue [4]. The prolonged increase in circulating blood volume can lead to myocardial hypertrophy and decreased compliance, in addition to the common comorbidity of systemic arterial hypertension. The combination of these cardiovascular alterations will eventually lead to an increase in left ventricular stroke work with a higher risk of arrhythmias, cardiac failure, or even sudden cardiac death [5-8].

Central to the pulmonary pathophysiology associated with obesity is an increased work of breathing due to a variety of factors including increased chest wall resistance, increased abdominal pressure altering diaphragmatic position, and even respiratory muscle dysfunction. In fact, many pulmonary function measurements are decreased in obese individuals including tidal volume, vital capacity, total lung capacity, and functional residual capacity. However, despite lower lung volumes, obese patients actually have higher minute ventilation (due to an increased respiratory rate) in an attempt to compensate for an associated increase in oxygen consumption and carbon dioxide production [2,3,9].

Two other common pulmonary comorbidities, which may impact the care of obese surgical patients, include the Obesity Hypoventilation Syndrome and Obstructive Sleep Apnea. The Obesity Hypoventilation Syndrome, usually seen in the super-obese, is characterized by hypercapneic respiratory failure and alveolar hypoventilation. The clinical implication of this syndrome is a worsening of the chronic hypoxia and hypercapnia of obesity leading to even higher minute ventilation. In severe cases the extreme pulmonary compromise associated with Obesity Hypoventilation Syndrome may even lead to right heart failure [2]. Though not usually as severe, Obstructive Sleep Apnea will complicate the care of the obese surgical patient. Sleep apnea occurs from narrowing of the upper airway due to excess adipose deposition. This narrowing is worsened during sleep or in the recumbent position. Obstructive Sleep Apnea may increase the risk of respiratory failure [especially in the postoperative period] or extend the need for mechanical ventilation [10].

The Metabolic Syndrome of obesity (known by a variety of other names including Syndrome X, Multiple Metabolic Syndrome, Insulin Resistance Syndrome, Deadly Quartet, DROP Syndrome) is beginning to surface as a major underlying factor in the physiology and pathophysiology of obesity. Though definitions of the Metabolic Syndrome vary, the clinical manifestations include abdominal obesity, dyslipidemia, hypertension, insulin resistance, and proinflammatory as well as prothrombotic states [11]. Though a detailed discussion of the Metabolic Syndrome is beyond the scope of this review, this constellation of the metabolic abnormalities will carry far-reaching implications on the care of the obese surgical patients, and surgeons should develop a fundamental understanding before caring for this unique population of patients.

## Trauma

The ramifications of obesity as it relates to the care of trauma patients have been relatively ignored in the literature until recently. A few studies in the early 1990's investigated the effects of obesity in trauma patients with conflicting results. Morris et al used a case-control design to study the effects of a variety of pre-existing medical conditions (including obesity) on in-hospital mortality of trauma patients [12]. They matched 3,074 trauma deaths with 9,869 trauma survivors and found that cirrhosis, congenital coagulopathy, ischemic heart disease, chronic obstructive pulmonary disease, and diabetes all significantly increased the risk of dying after trauma. However, obesity was not identified as a risk factor for death. Similarly, a study by Milzman et al prospectively investigated pre-existing diseases on trauma outcomes [13]. This study collected 7,798 trauma patients (13% of whom were obese) and found obesity was the only preexisting disease not associated with worse outcomes after trauma. One of the earliest studies to specifically address obesity and trauma found conflicting data [14]. Choban et al retrospectively reviewed 184 blunt trauma patients (10% obese) and found obese patients had a significantly higher mortality as well as an increased number of pulmonary complications.

More recent studies have corroborated the results of Choban's paper, implicating obesity as an adverse factor for injured patients. In 2004 Neville et al evaluated outcomes in severely injured blunt trauma patients [15]. They examined 242 severely injured blunt trauma patients (26% obese) and found that obese patients more often developed multiple organ failure and had a higher mortality. In fact, obesity was associated with a six-fold increase in mortality following severe blunt trauma. In a follow up study from the same institution, a larger cohort of blunt trauma patients was examined [16]. The follow-up study included 1,153 blunt trauma patients, with 283 (25) obese patients. Once again obesity was associated with an increase in complications and obesity was an independent risk factor for in-hospital mortality. In another large series, Byrnes et al found that obese trauma patients were significantly more likely to experience complications and death than their lean counterparts [17]. Another interesting contribution was a study aptly entitled "The Cushion Effect", which evaluated outcomes after motor vehicle crashes comparing lean, overweight, and obese patients [18]. This study found that overweight patients were actually protected from dying during a motor vehicle crash, while obese patients once again had higher mortality when compared to lean patients.

An intriguing development from recent research involving obesity and trauma has been the identification of differences in injury patterns between obese and lean trauma patients. In 1992, Boulanger et al compared injury patterns between obese and lean individuals who sustained blunt trauma [19]. They found that obese blunt trauma victims were more likely to suffer pulmonary contusion as well as pelvic, extremity, and rib fractures. However, the obese patients less often sustained head and liver injuries. Very similar results were reported by Brown et al, who found severely injured obese blunt trauma patients had fewer and less severe head injuries and more chest injuries and lower extremity fractures [16]. Similarly, Arbabi et al found that obese motor vehicle occupants sustained more severe lower extremity injuries [18]. Overall, it appears that obese patients sustain different injury patterns than lean patients after blunt trauma. In particular, obese patients seem to receive fewer and less severe head injuries, while sustaining more injuries to the thorax and extremities.

## Emergency surgery

With the combination of the obesity epidemic and the arrival of the "acute care surgeon" we must be prepared to care for critically ill obese surgical patients with ever increasing regularity. When dealing with the common emergency general surgery diseases in the obese patient, the basic principles of general surgery apply. However, diagnosis may be difficult or delayed in obese surgical patients due to unreliability of physical examination [20], inaccuracy of diagnostic ultrasound [21,22], or unavailability of CT scan in centers not equipped to routinely image the morbidly obese.

Once a diagnosis has been made, laparoscopy appears to be the treatment of choice for patients requiring appendectomy or cholecystectomy. In a randomized trial Enochsson et al [23] compared open and laparoscopic appendectomy in overweight patients. They found that the laparoscopic approach was associated with less postoperative pain and a faster postoperative recovery. Multiple studies [24,25] have evaluated laparoscopic cholecystectomy in obese patients, and have found the laparoscopic approach to be comparable or better than the open approach for cholecystectomy. While the laparoscopic approach seems appropriate for appendectomy or cholecystectomy in obese patients, the same may not hold true for laparoscopic colectomy. Pikarsky et al [26] evaluated 162 patients [19% obese] undergoing laparoscopic segmental colon resection. They found obese patients had more conversions to open operation as well as more complications, leading to prolonged hospital stays in the obese patients.

No matter the operation performed the surgeon must expect an increased incidence of infectious complications in obese surgical patients. In particular, obesity has long been associated with an increased risk for wound infections following ulcer surgery, cholecystectomy, and hysterectomy [27]. More recently Choban et al studied 849 patients [21% obese] undergoing surgical procedures and found obese patients experienced more infectious complications including wound infection, bacteremia, and pneumonia [28]. Of interest, the increase in infectious complications for obese patients was seen only in clean and clean-contaminated cases, but not in contaminated or dirty cases. In a similar study, Canturk et al [29] evaluated infectious complications in 395 patients undergoing general surgical procedures. They found obesity was associated with an increased incidence of nosocomial infections; specifically more wound, urinary tract, and pulmonary infections. Once again, the increased infections of obese surgical patients only occurred in the clean and clean-contaminated cases.

## Surgical critical care

Though critically ill patients of all types require a significant amount of time and effort from the medical team, obese patients admitted to the surgical intensive care unit (ICU) present a particularly challenging population. Management of any critically ill patient will begin with airway assessment and management. Obese patients may provide a difficult airway to even the most experienced intesivists, and appropriate preparations are needed prior to establishing a definitive airway in an obese patient. Though some conflict exists, obesity is generally considered a risk factor for a difficult airway [30,31]. Despite difficulties most obese patients should be able to be orotracheally intubated. However, salvage techniques such as a laryngeal mask airway and awake fiberoptic intubation may be necessary [32]. The salvage airway for a surgeon, a cricothyroidotomy, may prove difficult in an obese patient due to a large neck, deep position of the trachea, distorted anatomy and inability to use standard length tracheostomy tubes.

Once a definitive airway has been established issues with mechanical ventilation must be considered, keeping in mind the deranged pulmonary physiology of obese patients mentioned previously. When placing an obese patient on the ventilator calculations of tidal volume should use ideal body weight rather than actual weight in order to minimize barotrauma or volutrauma. As obese patients present with chronic hypoxia and hypercapnia, simple measures such as reverse Trendelenburg positioning, positive end-expiratory pressure, or continuous positive airway pressure in the sleep apnea patient may improve pulmonary function [33,34]. Obesity seems to be associated with adverse pulmonary outcomes in the surgical ICU as evidenced in the study by Brown et al, which found that trauma patients admitted to the ICU more often had ARDS, required two more days of mechanical ventilation, and more often failed attempted extubation [16].

As mentioned previously, the metabolic syndrome of obesity will lead to a prothrombotic state and an awareness of the thromboembolic predisposition of obese surgical patients will allow the surgeon to take appropriate steps in an attempt to prevent deep venous thrombosis. Unfortunately, trying to prevent thromboembolic complications in obese surgical patients can be difficult at best. First of all, many of the mechanical devices are not designed to fit obese patients [10]. Most obese patients cannot wear sequential compression devices for the calves and thighs as well as TEDs™. Sequential compression devices for the feet are more easily applied to the obese patient, but their efficacy is unknown. More recently, low molecular weight heparin has become the standard thromboembolic prevention tool in surgical patients, using either a daily or twice daily weight-based dose. As low molecular weight heparins become more well understood, it appears "standard" dosing regimens are not appropriate for obese individuals, and higher doses may be required in obese surgical patients in order to achieve therapeutic anti-Xa levels [35,36].

When dosing medications in the ICU the appropriate weight for dosing can vary significantly depending on the medication. Consultation with an ICU pharmacist is essential to determine whether actual body weight, adjusted or dosing body weight, or ideal body weight should be used to dose a particular medication [3]. When considering nutritional needs of the critically ill or injured obese patient, the surgeon must be aware of the paradoxical response of obese individuals who experience metabolic stress. Rather than using abundant fat stores, obese patients will preferentially metabolize protein during the stressed state [37]. This counterintuitive metabolic response by critically ill obese patients will influence nutritional decisions in the ICU.

## Conclusion

A great deal more research is needed to investigate the influence obesity imparts on the care of trauma and emergency surgery patients. However, a few reasonable conclusions can be drawn from the currently available literature. Obese blunt trauma patients sustain different injuries than lean patients. In particular, obese patients suffer fewer and less severe head injuries and more thoracic and extremity injuries. Despite fewer head injuries, obesity seems to impart an adverse effect on outcomes after injury. Obesity presents a diagnostic challenge fort the emergency surgeon. With the exception of colorectal procedures, laparoscopy appears to be the treatment of choice for obese patients with common surgical diseases such as appendicitis and cholecystitis. Following general surgery procedures, obese patients are at higher risk of postoperative infections. Obese surgical patients admitted to the ICU will present unique challenges for the surgeon with regards to airway management and mechanical ventilation, and pulmonary morbidity is to be expected. As part of the metabolic syndrome, the obese surgical patients present in a hypercoagulable state. Every effort should be made to prevent thromboembolic complications in this high-risk population, and for obese patients the prevention strategy should include higher doses of low molecular weight heparin and monitoring of anti-Xa levels. Particular attention should be paid during pharmacological and nutritional decisions when dealing with obese surgical ICU patients, as they require more sophisticated adjustments based on weight and degree of illness.

## Competing interests

The author(s) declare that they have no competing interests.

## Authors' contributions

CVRB participated in the conception, design, and coordination of the study. GCV participated in the conception, design, and coordination of the study. All authors read and approved the final manuscript.
